# Colony Size Evolution and the Origin of Eusociality in Corbiculate Bees (Hymenoptera: Apinae)

**DOI:** 10.1371/journal.pone.0040838

**Published:** 2012-07-13

**Authors:** Enrique Rodriguez-Serrano, Oscar Inostroza-Michael, Jorge Avaria-Llautureo, Cristian E. Hernandez

**Affiliations:** Laboratorio de Ecología Molecular and Filoinformática, Departamento de Zoología, Facultad de Ciencias Naturales y Oceanográficas, Universidad de Concepción, Concepción, Chile; University of Leeds, United Kingdom

## Abstract

Recently, it has been proposed that the one of the main determinants of complex societies in Hymenoptera is colony size, since the existence of large colonies reduces the direct reproductive success of an average individual, given a decreased chance of being part of the reproductive caste. In this study, we evaluate colony size evolution in corbiculate bees and their relationship with the sociality level shown by these bees. Specifically i) the correlation between colony size and level of sociality considering the phylogenetic relationship to evaluate a general evolutionary tendency, and ii) the hypothetical ancestral forms of several clades within a phylogeny of corbiculate bees, to address idiosyncratic process occurring at important nodes. We found that the level of social complexity in corbiculate bees is phylogenetically correlated with colony size. Additionally, another process is invoked to propose why colony size evolved concurrently with the level of social complexity. The study of this trait improves the understanding of the evolutionary transition from simple to complex societies, and highlights the importance of explicit probabilistic models to test the evolution of other important characters involved in the origin of eusociality.

## Introduction

Bourke [1] proposed that colony size, along with kin structure, is one of the main determinants of complexity in insect societies. This hypothesis is based on the reproductive conflict between members of a colony: any change in colony size entails a direct consequence for the reproductive potential of a worker. When colony size increases, workers experience a loss of reproductive power, given a decreased chance to be part of the reproductive caste, or to replace the queen. The main mechanisms involved in lowering the reproductive potential of a worker, are the diluted by number process and worker policing [2]. As colony size increases, the proportion of any worker’s eggs among worker-laid eggs falls because it becomes increasingly difficult for the worker to prevail over all others and the mutual prevention by workers of another’s reproduction increases [1]. Bourke [1] has shown that these mechanisms hold for different queens’ mating frequencies and all ploidy systems, with a few exceptions requiring further explanations (see [2]–[4]). As a consequence, the behavioral/morphological divergence between workers and reproductives is the result of strong selection for specialized workers with low reproductive potential [5].

A simple contrast between the level of sociality and colony size of different groups of social insects provides a positive relationship between these variables (*e.g.* [6]–[10]). However, standard correlation statistical tests are not suitable for use with non-independent data, such as species with shared phylogenetic history [11], [12]. Furthermore, application of “phylogenetic corrected” statistical tests (a correlation between variables to which have been removed the phylogenetic component of variance, such as Phylogenetic Independent Contrasts; [11]), only gives information about the actual relationship between these variables [11]. However, other tools of the comparative phylogenetics method [12], allow us to explore at least two aspects of Bourke’s hypothesis in a explicit phylogenetic context: i) if the correlation between colony size and level of sociality is implemented considering the phylogenetic relationship, the associative pattern could shown a general evolutionary tendency, and ii) the hypothetical ancestral forms of several clades within a phylogeny of social insects could be informative about idiosyncratic processes occurring at important nodes, and possibly why colony size evolved concurrently with the level of social complexity (*e.g.* [13], [14]). The latter approach has been used to evaluate the origin of monogamy and the correlation of this trait with both, cooperative breeding and eusociality (in birds, [15]; and Hymenoptera, [16] respectively).

The subfamily Apinae (also “corbiculate bees” because they possess a “corbicula”, structure in the metatibia that enables the transport of pollen) is a monophyletic group, presenting sociality levels that include solitary species, to communal (Euglossini), to eusocial species with diverse caste determination strategies. The tribe Bombini shows a behavioral worker caste and subtle morphological divergence between reproductives and workers. The Apini and Meliponini tribes have morphologically distinct reproductives and workers [17]. Also, corbiculate bees show great variation in colony sizes, ranging from solitary or few individuals in Euglossini, to dozens of thousands in Apini [18]. These bees therefore represent an excellent model for studying the correlation between colony size and complex societies through the use of explicit evolutionary models [19]. Recently, Cardinal *et al.* [20], propose a novel, detailed, comprehensive molecular phylogenetic analysis of all 33 apid tribes based on seven nuclear molecular markers. In this work, the obtained phylogenetic tree shows that Meliponini and Apini are not sister clades. Thus, “multiple origins” of complex sociality is the most probable evolutionary history of this trait based on the current phylogenetic hypothesis. Under this phylogenetic context, this group of Hymenoptera allows the direct testing of Bourke’s hypothesis given that there is no single character transition from solitary to eusocial species and thus any evolutionary transition in social system should be a consequence of an increase in colony size. In this study we assess two critical points of this hypothesis: a) the phylogenetic correlation between colony size and social structure, and b) the evolution of colony size and the social structure of the corbiculate bees through the reconstruction of ancestral character state.

## Results

### Phylogenetic Relationships of Corbiculate Bees

**Figure 1:**
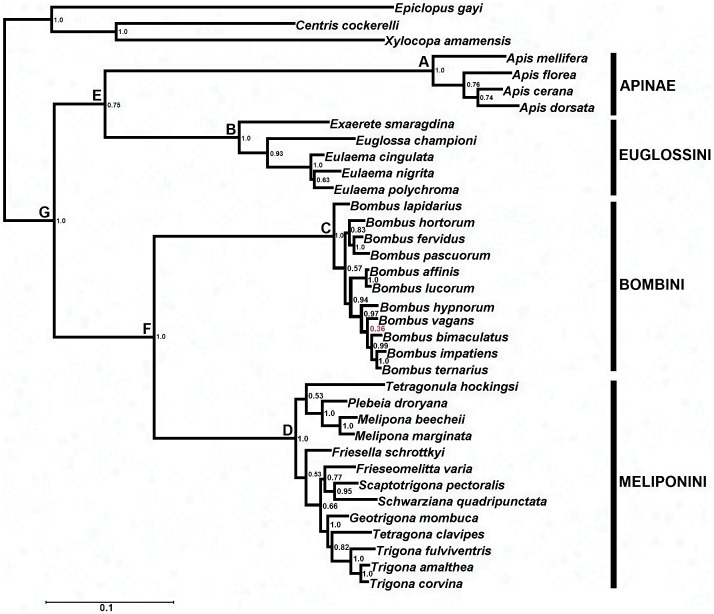
Phylogenetic reconstruction of corbiculate bees based on Long Wavelength Rhodopsin and Arginine Kinase. The tree was obtained by means of a phylogenetic mixture model based on Bayesian approach. The numbers next to nodes indicate the posterior probability of occurrence of the clade. The letters above the nodes correspond to the hypothetical ancestors, whose most probable character state is shown in Figures 2 and 3.

The phylogenetic reconstruction that best fit to the rates and patterns of molecular evolution of the data was that obtained with 2GTR+Γ matrices. The consensus tree topology showed corbiculate bees to be a completely sustained monophyletic group (Fig. 1, Node G), where each tribe corresponded to a highly supported natural group. The relationship between tribes was observed to have a higher posterior probability (Fig. 1, Node E and F). Our phylogenetic hypothesis has no differenced from that reported by Cardinal *et al.* [20], so is a good historical support for further comparative analysis.

### Colony Size and Social Structure Evolution

**Figure 2:**
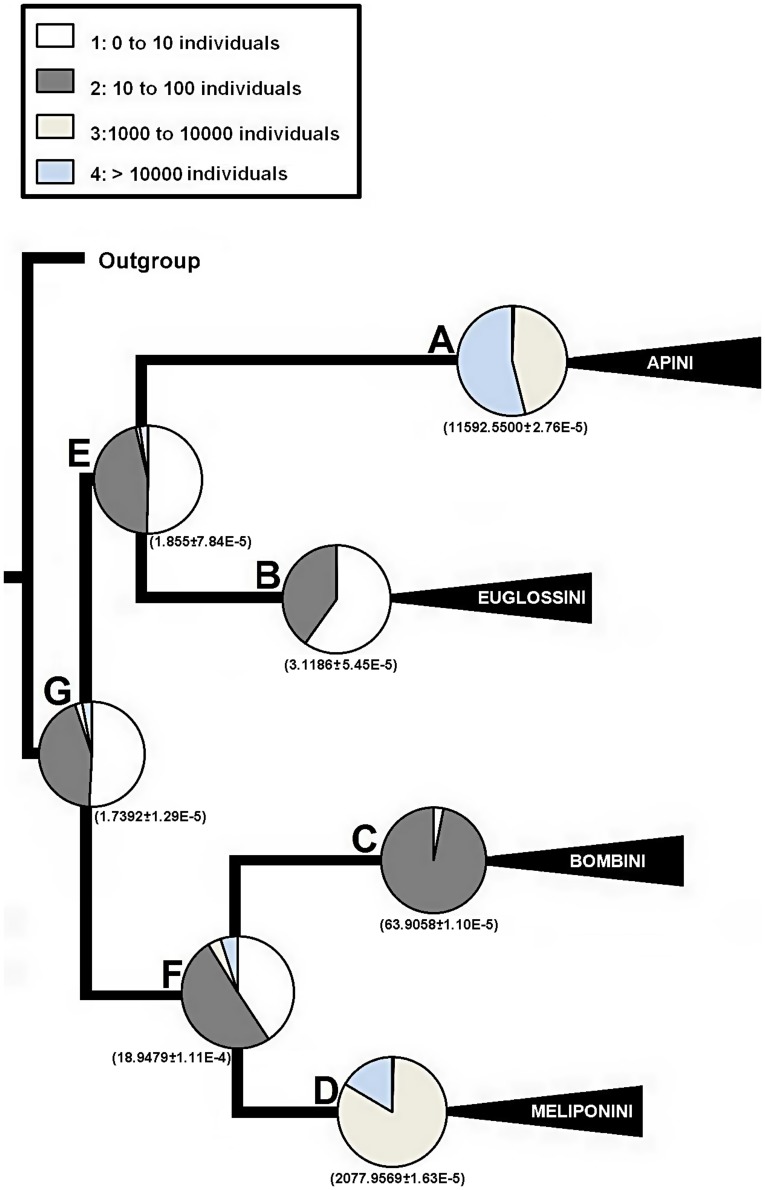
Reconstruction of the ancestral state of the colony size trait. The reconstruction was based on both topology and branch lengths of the Bayesian phylogenetic trees. In parenthesis is shown the mean value and standard error for the continuous character reconstruction.

**Figure 3:**
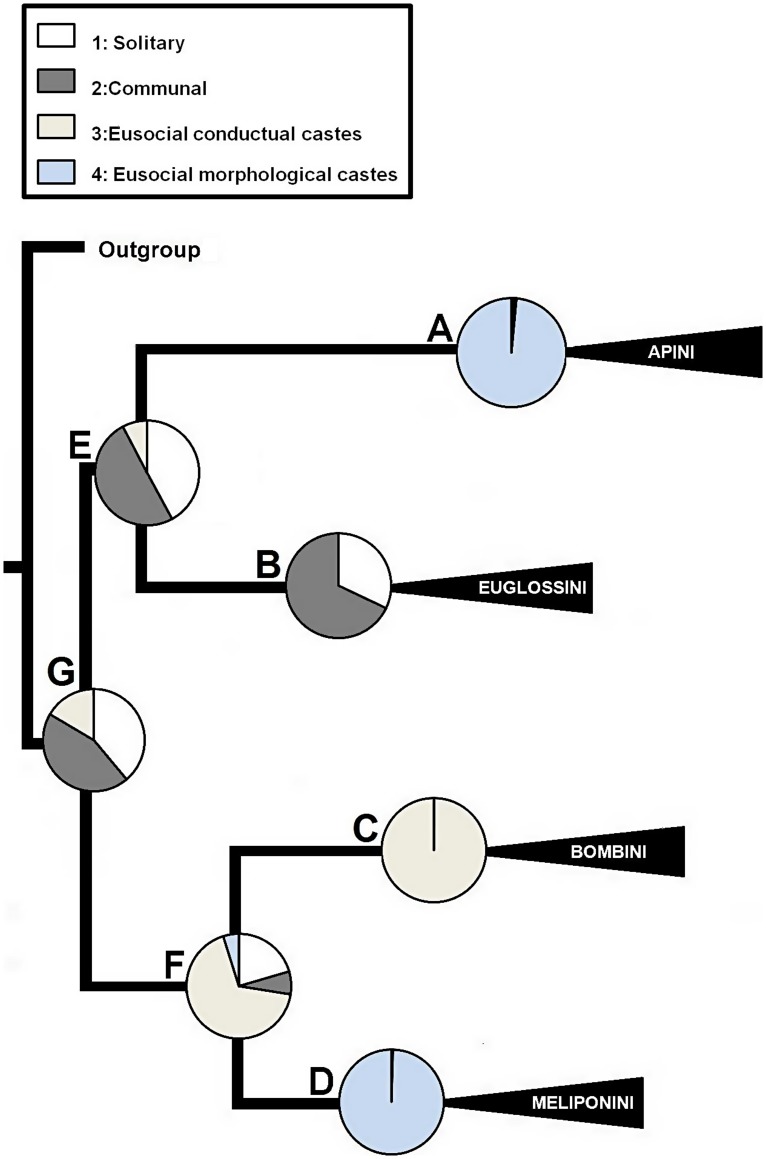
Reconstruction of the ancestral state of the level of sociality trait. The reconstruction was based on both the topology and branch lengths of the Bayesian phylogenetic trees.

The predominant evolutionary transition rates among the different colony size states were, q_12_, q_21_ (Table 1); that is, the transition from few to hundreds of individuals in both directions. In addition, the predominant transition rates in the evolution of sociality were q_21_, q_12_, (Table 1), that is, the transition from solitary to communal in both directions. The ancestral character state estimation for the phylogeny nodes indicated that the most probable common ancestor of the corbiculate bee tribes was a colony of few individuals (Fig. 2 Node G; probability (p) = 0.51; 1.7 individuals) with a communal sociality level (Fig. 3 Node G; p = 0.44). The ancestor of the Meliponini and Bombini has a higher probability of having tens/hundreds of individuals (Fig. 2 Node F; p = 0.51; 18.9 individuals) and eusocial with behavioral castes (Fig. 3 Node F; p = 0.68). On the other hand, the ancestor of Euglossini and Apini has more probability of having colonies with few individuals (Fig. 2, Node E; p = 0.50; 1.9 individuals) with a communal social structure (Fig. 3 Node E, p = 0.50).The development of morphological castes occurred independently in two lineages: In the Meliponini ancestor (Fig. 3 Node D, p = 0.99) and in the Apini ancestor (Fig. 3 Node A, p = 0.98). The ancestors of both tribes had large colonies (Fig. 2 Node A p = 0.54; 11592 individuals; and Node D p = 0.83; 2077 individuals).

**Table 1:** Ranked table of the mean instantaneous transition rate of character state estimated under the Markov k-state evolutionary model for discrete traits.

Trait	Transition Rate	Mean	4SE
**Colony Size**			
	q12	25.70	2.05
	q21	19.20	0.66
	q43	9.95	1.90
	q34	9.55	0.34
	q23	5.50	0.07
	q42	0.70	0.29
	q41	0.03	0.05
	q13	0.01	0.02
	q32	0.00	0.01
	q14	2.71E-06	5.91E-06
	q31	1.18E-06	2.39E-06
	q24	4.78E-07	8.34E-07
**Level of Sociality**			
	q21	11.64	0.25
	q12	6.97	0.14
	q13	4.85	0.20
	q34	3.92	0.14
	q24	1.09	0.20
	q23	0.81	0.05
	q32	0.43	0.04
	q31	0.22	0.07
	q14	Na	Na
	q41	Na	Na
	q42	Na	Na
	q43	Na	Na*

* Not applicable; set to 0.

Colony size (N° of individuals): **1:** 0 to 10; **2:** 10 to 100; **3:** 1,000 to 10,000; and **4:** over 10,000.

Level of Sociality: **1:** solitary; **2:** communal; **3:** eusocial with behavioral castes; **4:** eusocial with morphological castes.

Finally, the results of the phylogenetic logistic regression show that the effect of colony size on social structure is significantly different from zero if the evolutionary relationships of corbiculate bees are part of the model (Table 2). Larger values of colony size are strongly related to more complex social structure (*i.e.* eusocial with morphological castes; Table 2, b1 = 7.7475; p<0.001). Concordantly, smaller colony sizes are strongly related with the absence of complex societies (*i.e.* eusocial with behavioral/morphological castes; Table 2, b1 = −2.2793; p<0.001).

**Table 2:** Phylogenetic logistic regression parameter estimates for the effect of colony size on the social structure of corbiculate bees (b_1_). (a) Phylogenetic signal.

Parameters	Estimate^a^	Standard^a^ Error	Bootstrap^b^ mean	Bootstrap^b^ confidence interval	Bootstrap p value
**Presence (1)/absence (0) morphological caste versus colony size**					
A	−0.7695		−2.1676	(−4, 4)	0.3315
b_0_	−1.8484	1.6857	−1.5015	(−2.6069, 0.32882)	0.094
b_1_ (colony size)	7.7574	3.5615	6.3929	(3.0789, 7.7574)	**<0.001**
**Presence (1)/absence (0) of solitary social level**					
A	3.9999		3.3298	(−4, 4)	0.052026
b_0_	−1.2647	1.2826	−1.0006	(−2.4455, 0.65283)	0.26013
b_1_ (colony size)	−2.2793	0.73965	−2.0557	(−3.1875, −0.8914)	**<0.001**

a Parameters of logistic regression and standard errors of the estimates were obtained using the GEE approximation (see Ives & Garland, 2010).

b Parametric bootstrapping was performed by simulating 2000 data sets to obtain confidence intervals. Parametric bootstrapping was also used to test the null hypotheses that there is no phylogenetic signal in the residuals (H_0_: a = −4, 1-tailed test) and that the regression coefficients equal 0 (H_0_: b_i_ = 0, 2-tailed tests).

## Discussion

### Colony Size Evolution and the Origin of Eusociality

Our results, based on analyses that evaluated the phylogenetic uncertainty, and the reconstruction of ancestral states, show that the colony size of corbiculate bees coevolves with the level of sociality (Fig. 2, 3; Table 2). Colony size is a complex trait, determined by various factors that favor or restrict the number of individuals [1]. Those which restrict colony size are associated with traits related to life history [21], periodicity of trophic resources and space for nesting [22], among others. These multiple factors have differential importance throughout the tribes of Apinae. For example, with respect to the physical space for nesting [22]: the species in the Meliponini tribe prefer to use hollow trees for nesting, and there are also hypogeous species [23]. There is a strong spatially limiting factor involved in the development of highly large colonies, such as those of the Apini. Other factors could be influencing colony size in the Bombini tribe, especially factors correlated with latitude, due to the annual cycle of the colonies of this tribe [24], [25]. These ecological factors that determine the colony size could be responsible for the qualitative change from the most recent common ancestor (MRCA) of Apini + Euglossini (Fig. 2, node E: categorical 0 to 10; continuous 1.9 individuals) to the MRCA of Apini (Fig. 2, node A: categorical >10000; continuous 11592 individuals).

The lack of intermediate form between these nodes does not have a straightforward interpretation. Some insight can be obtained from allodapine bees (Apidae: Allodapini), in which the colony size and the level of sociality is a function of environmental and ecological variables in the facultative eusocial *Exoneurella tridentata* (*i.e.* a xeric habitat and durable nesting substrate; [26]). This bee is an exception within the tribe, given that it shows both a discrete morphological gap between queen and worker castes, and evolution of colony size (∼40 females) with accelerated tempo [26], [27]. The colony size and eusociality evolution of *E. tridentata* thus represent a discontinuity in the evolutionary change of the Allopadini tribe, which could be associated with overcoming a strong selective barrier (*sensu* [26]). A barrier that could be important at the MRCA of Apini + Euglossini (Fig. 2; node E), can be inferred from a general exploration of the main ecological traits of Euglossini vs Apini. Euglossini is a tribe of highly specialized bees on a few plant families that inhabits almost exclusively the Neotropics, whereas Apini is a tribe with a wide range of both food resources and distribution [18], [28]. For example, strong resource dependence has been detected in *Euglossa nigropilosa,* which preclude many communal nesting females [29]. This could imply that few individuals in a communal society (Fig. 2; node E) can either take advantage of generalist traits and increase colony size, or hold the character state as consequence of specialization (Fig. 2; nodes A, B). In the same way, the evolution from the communal MRCA of Apini + Euglossini (Fig. 3, node E) to the eusocial with morphological castes MRCA of Apini (Fig. 3, node A) can be addressed by scarce evidence from the halictine sweat bee *Halictus sexcinctus* (Hymenoptera: Halictidae [30]). This species shows a communal/eusocial polymorphism in the same population, where the eusocial colonies are composed of distinct morphological castes, which also differ from the communal foundresses [30]. Since the social polymorphism of *H. sexcinctus* is an extraordinary case within Halictidae, Richards *et al.* [30], argue that it may represent an unstable intermediate step in the evolution of social behavior. The consequences of this labile character state are either to promote some individuals to dominate reproduction or the founding of solitary colonies [30]. However, these species with facultative eusociality (*E. tridentata* and *H. sexcinctus*) show small incremental changes in colony size between the communal and eusocial states. The differential of colony size and level of sociality character states found on the transition from Node E to Node A (Fig. 2 and 3) seem to be quite different. Then, explanations are needed beyond actual ecological forces for this.

Boomsma [31], [32] proposed that lifetime monogamous species are more likely to evolve obligate eusociality (*i.e.* eusocial with morphological castes). This hypothesis argues that in lifetime monogamous species, relatedness is maximized throughout the lives of helper cohorts, promoting the evolution of sterile caste [32]. The previous examples of allodapine and halictid bees only show an advanced form of cooperative breeding [32]. Although this proposal has been supported in a phylogenetic context, including the estimated character state at MRCA of corbiculate bees [16], further questions arise. For instance, once lifetime monogamy is established, how do sterile castes then evolve? In this context, the explanation for the transition between the MRCA of Apini + Euglossini to the MRCA of Apini, could be addressed from the reproductive abilities of a worker “by its own choice” [5]. The direct reproductive benefits tend to decrease, as well as the indirect benefits increase, while the colony grows in size. This is because the worker loses opportunities to replace the queen in a colony where more and more workers are trying to take over the colony [5], and, concurrently, this crowded colony represents a full-sibling system. Then, the benefits may outweigh the costs simply by increasing the number of individuals in a colony. Bourke [1] used an extended argument that incorporates the mutual inhibition between workers of their reproduction potential (*i.e.* worker policing, [2]). This behavior leads to the selection of highly specialized workers accompanied by morphological skew between castes, which at the same time incurs positive feedback, permitting the existence of larger colonies. If this mechanism operates, then it is not necessary that the ancestor of eusocial forms with morphological castes be eusocial with behavioral castes, only that it have a monogamous reproductive system. Based on our results, we suggest that it is possible for eusociality with morphological castes to evolve either from a gradual increase in colony size and complexity (Fig. 2 from node F to node D), or from a threshold of colony size (Fig. 2 from node E to node A). Once it has reached this character state, the species can’t revert, consistent with previous studies [32] (Fig. 2 Node B, C).

Finally, our study, based on a Bayesian probabilistic framework, provides strong support for Bourke’s proposal [1]; since we have observed that the one of the main determinants in the evolution of morphological castes in complex societies of corbiculate bees is colony size. The study of this trait improves our knowledge regarding the evolutionary transition from simple to complex societies, and highlights the importance of explicit probabilistic models to test the evolution of eusociality and other important characters in social species.

## Material and Methods

### Ecological Data

We compiled a datebase of the colonial size and social structure of corbiculate bees from an exhaustive search in the literature (Table S1). Colony size data were treated both as categories following the ranges described by Bourke [1], and continuous values (*i.e.* as mean value reported in the literature (Table S1). For the discrete coding, four colony size categories were used: 1 = 0 to10 individuals; 2 = 10 to 100 individuals; 3 = between 1,000–10,000 individuals; and 4 = 10,000 or more Meliponini and Apini individuals. The continuous and discrete coding uses of colony size are proposed as complementary approaches given the missing categories (100–1000) in the data. For the case of the social system variable, we assign categorical variables to level of sociality as proposed by Kukuk [33], where 1 = solitary; 2 = communal; 3 = eusocial with behavioral castes; 4 = eusocial with morphological castes.

### BMCMC Molecular Phylogeny

Although the phylogeny of corbiculate bees is well resolved (see [20]), we reconstruct the phylogenetic relationships of the species belonging to this groups to obtain a sample of phylogenetic trees. With this sample of topologies and branch lengths, we can incorporate the phylogenetic uncertainty in the estimation of ancestral character states. We used DNA aligned sequence data from Cardinal *et al.* [20], specifically the Long Wavelength Rhodopsin (LWRh) and Arginine kinase (Argk) nuclear genes from the 33 species of corbiculate bees and three species of the sister group (*Centris cockerelli*) and from more distantly related families as outgroup (*Epiclopus gayi*, and *Xylocopa amamensis*; sensu Cardinal *et al.* [20]; Table S2). The alignment was performed using Clustal W [34]. These sequences were concatenated to reconstruct a phylogenetic hypothesis using the Markov Chain Monte Carlo (MCMC) method within Bayesian framework (BMCMC) to estimate the posterior probability of phylogenetic trees. Due to the markers selected comimg from different genomic locations and, as has been shown, their patterns and rates of nucleotide substitution being heterogeneous [20], we applied a general likelihood-based mixture model (MM) of gen-sequence evolution as describes by Pagel and Meade [35], [36] based on General Time Reversible model (GTR, [37]). The MM model accommodates cases in which different sites in the alignment evolved in qualitatively distinct ways, but does not require prior knowledge of these patterns or partitioning of the data. The Reversible-Jump Markov Chain Monte Carlo (RJMCMC) procedure [38], [39] was used for integrating the results of all patterns, and produces an MM that summarizes the sequence evolution. This approach enables researchers to explore the variety of possible models and parameters, converging towards the model that best fits the data in the posterior tree sample. These analyses were made in BayesPhylogenies 1.1 software (http://www.evolution.rdg.ac.uk/BayesPhy.html). We ran four independent BMCMC analysis using 35,707,000 iterations of the phylogenetic trees, sampled every 1000th to assure that successive samples were independent. We used the chains which had the highest marginal likelihood. From this sample of trees we removed the first 500 trees of the sample to avoid including samples before the convergence of the Markov Chain. Then we re-sampled every 46 trees to obtain a final sample of 770 trees with an autocorrelation of −0.008678 in ln-likelihood. This final sample was used for the comparative analysis.

### Estimation of Ancestral Character States, Evolutionary Transition Rates and Phylogenetic Logistic Correlation

We used the ancestral state reconstruction package available in BayesTraits 1.0 software [40], to evaluate the evolution of social traits and the colony size of corbiculate bees. For this we used both the topology as well as the branch lengths obtained from the sample of phylogenies in the BMCMC molecular phylogenetic analysis (in this case we used 770 trees). Using these data we were able to relate the evolution of eusociality, with estimated colony size ancestral states. For reconstruction of the ancestral characters states we used the continuous-time Markov k-state model [41], [42] estimating states under Bayesian MCMC framework. We run MCMC analysis with a uniform prior distribution on the rate coefficients with a range from 0 to 100. Several values of the Rate Deviation parameter were alternated to avoiding spurious acceptance between successive MC steps (*i.e.* appropriated acceptance values are in the range 0.28 to 0.32; Dr. Andrew Meade, personal communication). We ran 50000000 MCMC iterations, discarding the first 50000 iterations to avoid results outside the likelihood convergence. This analysis was run in the Multistate module of BayesTraits software [40]. We determined all instantaneous transition rates defined as q*_xy_*, where *x* is the initial character state at the beginning of a branch, and *y* the final state at the tip of the same branch [41]. These rates were estimated among the discrete states of the colony size character, for both forward and backward rates, but without establishing restrictions in the trait transitions, that is, we allowed this character to vary from a few to a large number of individuals and from a large number to a few, going through all the possible intermediate values in the different interior nodes of the phylogenies (Table 1). Concordantly, we estimated the ancestral character states of the continuous values of colony size (*i.e.* mean of colony size reported for each species incorporated in this study; Table S1). This analysis was run in the Continuous module of the BayesTraits software [40]. For this, we ran 50000000 MCMC iterations and discarding the first 50000 iterations. In this approach the Data Deviation parameter was treated in the same way that Rate Deviation parameter to achieve a 28–32% acceptance. In the same way we determined some transition rates for the level of society character evolution, with important restrictions, that is, we allowed this character to vary between immediate states (*e.g.* solitary to communal an vice-versa: q_12_ and q_21_). The transition rates from eusocial with morphological castes to any other state (*i.e.* q_43_, q_42_, q_41_) were set to 0, given that the eusociality with morphological castes is an evolutionary endpoint without reversion [32]. Also, and given the extremely unlikely scenario with evolution of eusociality with morphological castes from a solitary ancestor, we restrict the rate q_14_ to 0.

Finally, we perform phylogenetic logistic regressions with Firth correction to evaluate the relationships between colony size and social structure through the evolutionary history of this group. Briefly, this method allows the analysis of cases in which the dependent variable is binary (0 or 1) and the values are non-independent among species, with phylogenetically related species tending to have the same value of the dependent variable. The method is based on an evolutionary model of binary traits in which trait values switch between 0 and 1 as species evolve up a phylogenetic tree [43]. So, to evaluate if higher colony size was correlated with higher level of sociality (*i.e.* morphological castes), we categorized the presence of morphological castes as 1 and its absence as 0 in the species under study. In addition, in order to evaluate the Bourke’s [1] prediction of that small colonies should never be complex (*e.g.* show morphological/behavioral castes) we categorized the presence of the lower social level (i.e solitary and communal social level) as 1 and its absence as 0. These regressions were performing using a variance-covariance matrix of the species trait constructed using the consensus of tree samples (previous section) in the module PDAP [44] of Mesquite software version 2.6 [45]. The phylogenetic logistic regression was run using the PloGReg.m function written by Ives and Garland [43]. This function simultaneously tests for phylogenetic signal while estimating the parameters of the regressions. The independent variable was standardized to have mean equal to zero and standard deviation equal to one; this makes the regression coefficients represent effect sizes of the independent variables whose magnitudes reflect the size of effect of the variable [43]. A bootstrapping procedure involving 2000 simulations (as proposed by Ives and Garland [43]) was used to generate the confidence intervals and test for statistical significance of the slope of the regression model and phylogenetic signal of the discrete data. Convergence of model parameters was achieved in all cases after these simulations.

## Supporting Information

Table S1 **Colony size and level of social complexity for the species used in phylogenetic comparative analyses.** Also are provided the reference for each data.
(PDF)

Table S2 **GenBank Access Number for the nuclear genes of each species used in this study.**
(PDF)
