# Supplementary material for: Colony Size Evolution and the Origin of Eusociality in Corbiculate Bees (Hymenoptera: Apinae)
Source: PLoS One. 2012 Jul 13;7(7):e40838. doi: 10.1371/journal.pone.0040838 (PMC3396608; doi:10.1371/journal.pone.0040838)
Supplement: Table S1 — Colony size and level of social complexity for the species used in phylogenetic comparative analyses. Also are provided the reference for each data. (PDF) [file pone.0040838.s001.pdf]

Table S1. Species used in this study and ecological data used in comparative analyses.

Taxa		Ecological Traits		
**Ingroup**	**Tribe**	**Colony Size**	**Sociability Level[1]**	**Reference[2]**
Apis cerana	Apini	6271	Eusocial with morphological castes	1
Apis dorsata	Apini	6884	Eusocial with morphological castes	1
Apis florea	Apini	36630	Eusocial with morphological castes	1
Apis mellifera	Apini	80000	Eusocial with morphological castes	2
Bombus affinis	Bombini	100	Eusocial with behavioral castes	3
Bombus bimaculatus	Bombini	10	Eusocial with behavioral castes	3
Bombus fervidus	Bombini	100	Eusocial with behavioral castes	3
Bombus hortorum	Bombini	120	Eusocial with behavioral castes	4
Bombus hypnorum	Bombini	31.4	Eusocial with behavioral castes	5
Bombus impatiens	Bombini	100	Eusocial with behavioral castes	3
Bombus lapidarius	Bombini	100	Eusocial with behavioral castes	3
Bombus lucorum	Bombini	100	Eusocial with behavioral castes	3
Bombus pascuorum	Bombini	100	Eusocial with behavioral castes	3
Bombus ternarius	Bombini	100	Eusocial with behavioral castes	3
Bombus vagans	Bombini	10	Eusocial with behavioral castes	3
Euglossa championi	Euglossini	4	Solitary	6
Eulaema cingulata	Euglossini	9.6*	Communal	7
Eulaema nigrita	Euglossini	9,6*	Communal	7
Eulaema polychroma	Euglossini	13	Communal	7
Exaerete smaragdina	Euglossini	1	Solitary	2
Friesella schrottkyi	Meliponini	1400*	Eusocial with morphological castes	8
Frieseomelitta varia	Meliponini	1200*	Eusocial with morphological castes	8
Geotrigona mombuca	Meliponini	2500*	Eusocial with morphological castes	8
Melipona beecheii	Meliponini	1192*	Eusocial with morphological castes	8
Melipona marginata	Meliponini	1330*	Eusocial with morphological castes	8
Plebeia droryana	Meliponini	2960*	Eusocial with morphological castes	8
Scaptotrigona pectoralis	Meliponini	4600	Eusocial with morphological castes	8
Schwarziana quadripunctata	Meliponini	1500*	Eusocial with morphological castes	8
Tetragona clavipes	Meliponini	7000*	Eusocial with morphological castes	8
Tetragonula hockingsi	Meliponini	Not Available	Eusocial with morphological castes	-
Trigona amalthea	Meliponini	10500*	Eusocial with morphological castes	9
Trigona corvina	Meliponini	22675*	Eusocial with morphological castes	9
Trigona fulviventris	Meliponini	8500*	Eusocial with morphological castes	9

***** Colony size values are the average of the values obtained from literature

[1] The classification of sociality level were obtained from [10]

[2] The references correspond to the sources from which the values of colonial sizes were obtained.

References

1. Seeley TD, Seeley RH, Akratanakul P (1982) Colony Defense Strategies of the Honeybees in Thailand. Ecol Monogr 52: 43-63.

2. Michener CD (2000) The Bees of the World, Johns Hopkins University Press. 972 p.

3. Boomsma JJ, Kronauer DJC, Pedersen JS (2009) The evolution of social insect mating system. In: Gadau J, Fewell J editors. Organization of insect societies: from genome to sociocomplexity. Harvard University Press. 617 p.

4. Williams R (2006). British bumblebees, their descriptions, life-styles and plants hosts, with particular reference to the old county of Somerset (vice-counties 5 & 6): an accumulation of information from a variety of sources. Venellus. Great Britain.78 p.

5. Hammond RL, Keller L (2004) Conflict over male parentage in social insects. PLoS Biol 2(9): e248. doi:10.1371/journal.pbio.0020248.

6. Eberhard WG (1988) Group Nesting in Two Species of *Euglossa* Bees (Hymenoptera: Apidae). J Kans Entomol Soc 61: 406-411.

7. Nantes-Parra G, González VH (2000) Notas sobre el nido de *Eulaema polychroma* (Hymenoptera Apidae: Euglossini). Actual Biol 22: 83-90.

8. Tóth E, Queller DC, Dollin A, Strassmann JE (2004) Conflict over male parentage in stingless bees. Insect Soc 51:1–11.

9. Slaa EJ, Wassenberg J, Biesmeijer JC (2003) The use of field–based social information in eusocial foragers: local enhancement among nestmates and heterospecifics in stingless bees. Ecol Entomol 28: 369–379.

10. Kukuk PF (1994) Replacing the terms “primitive” and “advanced”: new modifiers for the term “eusocial”. Anim Behav 47: 1475-1478.
