# Supplementary material for: Colony Size Evolution and the Origin of Eusociality in Corbiculate Bees (Hymenoptera: Apinae)
Source: PLoS One. 2012 Jul 13;7(7):e40838. doi: 10.1371/journal.pone.0040838 (PMC3396608; doi:10.1371/journal.pone.0040838)
Supplement: Table S2 — GenBank Access Number for the nuclear genes of each species used in this study. (PDF) [file pone.0040838.s002.pdf]

Table S2. Species used in this study. Also the GenBank access numbers for the genes used in the phylogenetic analyses (Arginine kinase and Long-Wavelength Rhodopsin)

Taxon		GenBank Number	
**Ingroup**	**Tribe**	**ArgK**	**LW Rh**
Apis cerana	Apini	EU184832	EU184839
Apis dorsata	Apini	AY267178	AF091733
Apis florea	Apini	EU184831	EU184838
Apis mellifera	Apini	EF032397	AF091732
Bombus affinis	Bombini	AY739497	AY739451
Bombus bimaculatus	Bombini	AY739500	AY739456
Bombus fervidus	Bombini	AF492863	AF492997
Bombus hortorum	Bombini	AF492853	AF492987
Bombus hypnorum	Bombini	EF032401	AF493013
Bombus impatiens	Bombini	AF492875	AF493009
Bombus lapidarius	Bombini	AF492871	AF493005
Bombus lucorum	Bombini	AY739522	AF493021
Bombus pascuorum	Bombini	AF492867	AF493001
Bombus ternarius	Bombini	AF492912	AF493046
Bombus vagans	Bombini	AY739537	AY267158
Euglossa championi	Euglossini	EU421629	AJ581740
Eulaema cingulata	Euglossini	EU421633	AJ581728
Eulaema nigrita	Euglossini	EU421685	AJ581732
Eulaema polychroma	Euglossini	EU421706	AJ581730
Exaerete smaragdina	Euglossini	EU421585	AJ581738
Friesella schrottkyi	Meliponini	EU163020	FJ042401
Frieseomelitta varia	Meliponini	FJ042200	FJ042403
Geotrigona mombuca	Meliponini	FJ042204	FJ042407
Melipona beecheii	Meliponini	FJ042225	FJ042428
Melipona marginata	Meliponini	EU163072	FJ042399
Plebeia droryana	Meliponini	FJ042252	FJ042455
Scaptotrigona pectoralis	Meliponini	FJ042263	FJ042465
Schwarziana quadripunctata	Meliponini	FJ042270	FJ042472
Tetragona clavipes	Meliponini	FJ042287	FJ042490
Tetragonula hockingsi	Meliponini	DQ813084	DQ813241
Trigona amalthea	Meliponini	DQ813070	DQ813227
Trigona corvina	Meliponini	EU049760	EU049805
Trigona fulviventris	Meliponini	EU049762	EU049807
**Outgroup**			
Centris cockerelli	Centridini	AY267180	AY267164
Epiclopus gayi	Ericrocidini	EU184833	EU184840
Xylocopa amamensis	Xylocpnini	AY267179	AY267163
